# 1. The British and Foreign Medico-Chirurgical Review. 2. The London Lancet, for January and February. 3. The True Pathological Nature of Cholera, and an Infallible Method of Treating It. By Geo. Stuart Hawthorne, M.D. 4. On the Cryptogamous Origin of Malarious and Epidemic Fevers. By J. K. Mitchell, A.M., M.D., Professor of Practical Medicine in Jefferson Medical College

**Published:** 1849-05

**Authors:** 


ART. IV.—1. *The British and Foreign Medico-Chirurgical Review.*
2. *The London Lancet*, for January and February.
3. *The true Pathological Nature of Cholera, and an Infallible Method of treating it.* By GEORGE STUART HAWTHORNE, M.D.
4. *On the Cryptogamous Origin of Malarious and Epidemic Fevers.* By J K. MITCHELL, A.M., M.D., Professor of Practical Medicine in Jefferson Medical College. Philadelphia: Lea and Blanchard. 1849.

In our April number we endeavored to give a brief historical sketch of cholera, and to notice its leading phenomena for the purpose of ascertaining its cause, in order to pave the way to an inquiry as to the best sanitary regulations for its prevention. In the present article we shall examine those measures that have been adopted in various parts of the world, as the best means for arresting the ravages of cholera, and shall enter upon an investigation of the most philosophic method of treatment for the disease.

We think we have clearly established the fact that cholera is exclusively a malarious disease. Of the truth of this view we have no kind of misgiving or dubiety, and if it be true, the means for preventing the ravages of cholera are very accessible to the public. A long series of observations, made in various parts of the world by a great number of philosophic physicians, have clearly established the operations of malaria, and the unvarying laws of that agent of destruction, and by taking advantage of this knowledge, we shall easily control its evil effects.
Among the important and essential sanitary measures for preventing the production of malaria, cleanliness stands conspicuous. Nature has written so legibly on the great value of cleanliness as a hygienic agent, that proof of the fact would seem to be almost unnecessary. But daily observation tells us, in language not to be misunderstood, how little the dictates of nature on this subject are heeded, and how constantly men appear to choose disease rather than health. The ravages of cholera have impressed these things, however, so forcibly upon the mind of the world, that there is reason to hope that greater attention will be paid to the subject of cleanliness hereafter. An immense improvement has taken place throughout England in all things pertaining to public health, and many places formerly noted for their accumulations of filth, have, under the influence of drainage, good sewerage, and an enlarged and scientific view of the subject, become clean and healthy.

Throughout the history of epidemic cholera we have found it hanging its dark pall over the abodes of filth, and expending its energies of destruction upon squalid wretchedness and discomfort, most generally. Wherever malaria most readily forms, there cholera commences its operations, and lingers amidst the atmosphere that is vitiated by the combined action of solar heat and moisture in the decomposition of vegetable matter. We trace Cholera, by its usual history, in the Gangenetic Delta, and especially in the tracts of country bordering on the Hoogley and

Jellinghey rivers. Its ravages along the banks of the Ganges and its tributary streams were awful, and in the summer season malaria usually shows itself in these regions upon a gigantic scale. Thus we find epidemic cholera in Delhi in July, in Bombay in August, Madras in October, and Ceylon in December. It commenced in Astracan in September, Orenberg in July, and at Astracan again in the same month. In September we find it in Moscow, in June at St. Petersburg and Archangel, in Riga and Dantzic in May, in Hungary in August, in Constantinople in July, in a portion of Greece in November, in Berlin in August, Vienna in September, and in Hamburg in October. In the Spring of 1832 cholera showed itself in Dublin, Belfast, Cork and other places in Ireland. In June 1832 it ravaged Montreal and Quebec, but had previously showed itself in many villages in Canada. In July it assailed New York; in August it was in Philadelphia, Baltimore, Washington, Cincinnati and New Orleans. Boston being almost entirely free from malaria has been almost untouched by cholera, and a similar exception has generally been observed throughout New England. Naples established the most rigid quarantines, and maintained them with sleepless vigilance, but in 1836 cholera showed itself in the malarious portions of the town, and ravaged them exclusively. Rome had a visitation in 1837, and Malta about the same time.

A critical examination of all the circumstances connected with these visitations of cholera indubitably establishes the existence of circumstances favorable to the production of malaria, as the immediate precursor of cholera, and to this fact, we do not know of the existence of a solitary exception.

On this subject, Dr. John Bell, who is very favorably known to the medical profession by his various and valuable publications on medical science, and who is an opponent of the doctrine of malaria, holds the following clear and forcible language: "The chief home and seat of cholera is in low, damp situations—on the banks of rivers, or near pools and ponds of water,—or which are encumbered with vegetable remains, and filth of any kind. Those parts of cities thus situated and circumstanced, have always suffered most, and some times have been the exclusive seats of disease. In all the chief cities of Hindostan, as in Calcutta, Madras, Bombay, Seringapatam, &c.: of Russia, as in Moscow, St. Petersburg, Astracan, of Germany, as in Vienna, Breslau, Berlin, Hamburg, of France, as in Paris and other places; of Great Britain and Ireland, as in London, Sunderland, Newcastle, Gateshead, Musselburgh, Dublin, Cork, &c., this fact has been placed beyond a doubt." The varying phases of seasons have been conspicuous in the history of cholera. In most places where the disease held a heavy and an unexpected hand, an unusual series of meteorological phenomena showed itself. Thus, in Lexington, Ky., a place celebrated for its healthiness, we find as the precursor of cholera in 1833, a state of things never known there before or since. In the midst of an unusually high temperature from early in May, up to the 4th of June there was almost a constant fall of rain, and early in June the cholera commenced, and raged until the hot dry weather in July and August dried up the moisture from the ground. Large cities that are unhealthy in wet summers escaped the cholera for years under the sanitary influence of unusually dry summers, and places that are sickly in dry summers, escaped by the fall of sufficient rain to cover the marshy surfaces of pestilential pools with water. Dr. John Bell says: "Many of the British physicians and surgeons in India describe frequent and great deviations from the usual order of the seasons before and during the existence of cholera; and they speak of unusually violent thunder-storms, 'violent squalls,' and storms of wind and rain."

In almost every instance we have examined, cholera commenced its ravages in the midst of filth, and has almost always lingered in such localities. Where there are exceptions to this fact, we shall find an explanation of the exception in some of the laws of malaria.

In the close of our former remarks, we referred to the invaluable lessons, given by nature in favor of cleanliness, and we shall now proceed to a detail of some most interesting facts on this subject. They speak a lesson that cannot be too strongly impressed upon the mind. Chambers, in his *Edinburgh Journal*, says: "Who that surveys the most ordinary landscape, unfitted perhaps to inspire the poet or awaken the imagination of the romancist, can point to any stain upon its smiling face? The fresh raiment of the fields, the hard features of the rocks, the stream descending in clear, sparkling, laughing, tumbling waters, or stealing in slower measure through the plain; the spotless aspect of the driven snow, the smooth laid surface of the sandy shore, the deep pellucid waters of the great ocean—these are all *clean*. There is no spot of filth to be seen in them, except where the purificatory process is actually going on. Then the heavens assume what we might perhaps consider a filthy aspect—the sky becomes clothed with sackcloth, the hills disappear in murky fogs, the mountain stream comes down in floods of mud, hurling along heaps of degraded materials; the sea casts up its mire and dirt, and at these times the law appears suspended; but, on the contrary, this is the very process itself by which the general result is obtained. In a little while all this seeming disorder ends, and the landscape only looks cleaner than ever when it is over. A vast practical benefit results from a chain of circumstances apparently so trifling as the gathering and discharging of a rain-cloud. All the impurities which a state of change necessarily entails, are thus removed; not only is the face of the earth renewed, and the crowding vegetation which luxuriates upon its fertile bosom reinvigorated, but is also washed *clean*, exposed afresh to atmospheric influences, while the gatherings of previous weeks are all swept down and deposited out of sight beneath the blue waves." * * * * * * "At first one would believe it almost impossible that a blade of grass, in immediate proximity as it is to a filthy soil, could be kept clean; the dirty splashing of a shower, or the down pressing influence of a breeze, would suffice to take all the beauty out of an artificial grass blade. How different the result! Pick a handful of the tender herb from the worst field, the very slushiest meadow, and it is found clean, fresh, shining, without a spot of dirt or any such thing, so that it looks as though it had just left the hands of the Great Artificer. This result is principally due to the lustrous coat of silex with which the blade is provided, and the polished, glittering surface of which denies attachment to a spot of dirt. Grass, however, is by no means the only class of plants furnished with a similar provision, a glazed surface, evidently intended principally for this end." * * * * "While we survey all nature, we may fully join in the expressions of Dr. Macculloch, and say that it presents that 'universal look of cleanliness and neatness, which is as striking as if there was a hand perpetually employed in no other office, preserving an order, which we cannot maintain in our possessions without constant labor.'

"Few minds will be found, we believe, which will resist the evidence here adduced to the existence of a law of cleanliness in creation; but if we turn to the animal kingdom, the testimony becomes quite conclusive. Many precautions against dirt in this, as in other divisions of nature, are *passive*. No one that looks upon the glittering corselet of a cockaroach, inhabiting, as it does, the dusty cracks and crannies of our kitchen floors all night, and spotless as it is, can deny the conclusion, that there is an admirable proviso against filth in this insect. And the same may be said of the metallic coated family of beetles; whose burnished backs repel alike the minutest speck of dirt or the heaviest peltings of a summer shower; and the wing-covers of these beautiful insects are, without doubt, while they are the shields, also the dirt-repellers of the delicate, gauze-like wings so artfully folded up beneath them."

"We probably judge rightly in supposing that the *active* demonstrations of cleanliness are the most interesting, and are likely to be the most impressive." These are made under the influence of processes of *combing*, *brushing*, *licking and washing*, and are so interesting in their character to those whose great province and pleasure it should be to observe very carefully all natural phenomena, that we feel that there is no necessity for an apology for a minute detail. If no direct lesson be taught of the great value and extent of the law of cleanliness, these details may awaken in the mind of the physician a sense of how much valuable information may be gleaned by a close observation of the walks of creation in all the departments of his profession. From an extended survey of animal life Cuvier was able to deduce that great and important law, which is of such constant use to the physician who knows how to apply it:—that animals possess vigor in a direct ratio with the amount of oxygen they consume. Nor is there any fact in nature that may not be useful to the physician. He should not permit any of the processes of nature to be too high for his reach; none of them should be looked upon as too humble for his investigations. Let us now look at some of the operations of nature under the law of cleanliness, and have our minds impressed properly with the value of this great hygienic principle.

On the subject of *combing* among the insect tribes, Chambers says: "One of the commonest and most curious examples of combing, for the purpose of cleanliness, may be observed by closely watching a common garden spider.

These insects are particularly exposed to dirt; the dust of the air, particles of their webs, or defilement from their prey, become entangled in the hairs of their legs, and would probably both materially add to the discomfort and to the disability of the insect for its active life, were they not removed. The wants of the creature have not been forgotten, and its mouth is furnished with serratures like the teeth of a comb. The insect puts its leg into its mouth, and gradually draws it through these teeth, so as entirely to comb off every particle of dust and dirt, which it then collects into a pellet, and carefully tosses away! In order that this operation may be thoroughly done, and no part of the leg escape, a little curved hook is added, which bends down over the edge of the comb, rendering the escape of any part of the leg impossible. This curious fact was first noticed by Mr. Rennie, who mentions it in an interesting paper published at the Royal Institution. "The bird well known as the fern-owl, or night-jar, has an instrument on purpose to effect this object, a real comb. One of its claws differs from all the rest in length, and in the remarkable fact of its being serrated or toothed like a comb; and such is the intention of the contrivance. It was long mistaken for an instrument with which to wound its prey. Other naturalists perceiving its resemblance to a comb, and considering the whiskers of the bird, conceived that it was intended to comb the bird's whiskers. But against this ingenious hypothesis it must unfortunately be mentioned, that some of the species possess the comb without the whiskers, in which case its function must be, on that supposition, unnecessary. The celebrated Alexander Wilson, the ornithologist of America, decided the question by finding in the "Whip-poor-will," a bird belonging to the same group, and the inner edge of one of the claws of which is also pectinated, portions of down adhering to the teeth. He therefore very rationally concludes that this instrument is most "probably employed as a comb to rid the plumage of the head of vermin, this being the principal, and almost the only, part so infested in all birds." In another portion of that splendid work, he mentions that the night-heron, or "qua-bird," possesses also a pectinated or comb-like claw, which has from thirty-five to forty teeth, and is used for a similar purpose to that in the last case mentioned.

"Under the head of combing, we are doubtless to include what is called the 'preening,' or, more correctly perhaps, the pruning of birds. Probably no creatures are more attentive to personal neatness than the generality of birds, and this they principally effect by embracing their feathers with the beak, then drawing the beak to the extremity, by which means all dirt and soil are speedily removed. In this healthy exercise it has been well said they have been 'commanded to delight,' for while it is a sanitary act, it is also one which seems to afford them great gratification. Were it not that this beautiful part of creation is always thus employed, what filthy objects would many become who have to seek their food in mud or in the earth! But, as Drayton has said, they are always

> Pruning their painted breasts; and thus under the most disadvantageous circumstances, the lustre of the bird of paradise, or the snowy purity of the swan, is never to be seen dimmed by dust or defiled by mud. Still, under the "combing" we may mention the most familiar example of all, the common blowfly. Who that has watched the ludicrous care with which this insect attends to its personal appearance, has not been reminded of human actions? When we remember our own maneuvers with the clothes brush, and compare them with those of the fly dusting *his* jacket, the action has all the oddity of a caricature. How carefully he sweeps down the wings, and then his eyes and head, as if he were on the very point of presenting himself at court, or to the consideration of some fair friend! The microscope reveals his instrument. It consists of two rounded combs placed at the bottom of the foot, and consisting of two or three rows of teeth, somewhat like a curry-comb; and this contrivance perfectly removes all extraneous matters, so that the cleanly insect flies off a complete beau, if luster and absence of dirt would constitute one.

"*Brushing* is the next division. The bee gives us a good example in point. This unwearied insect, in her perpetual search for honey, has to penetrate many flowers which abound in pollen or farina—the light delicate powder produced by the anthers of flowers. When she comes home, she looks quite an altered character, all dusty as she is with yellow pollen, so that she could scarcely be recognized as the modest brown insect which the morning saw depart from the hive. The principal cause of this is the hairiness of her body, the pollen particles sticking fast in the pile. The insect stops and raising her hind-legs, which are set with thick hairs, she brushes every particle clean off, but as the pollen is valuable, she does not throw it away; on the contrary, she kneads it into little masses called bee-bread, and then enters the hive, having stowed it away in certain little pockets behind. Many spiders are provided with brushes of close-set hairs which effect the same purpose; and the foot cushions of the cat must be considered as instruments of similar intention. We are often presented with examples of *licking* as an operation of this kind. The cat takes incessant pleasure in it, and is very particular about her children too, whom she licks continually when they are young. Other animals have similar propensities, and hence arose the popular myth about the bear licking her cubs into shape, when she was, in fact, only giving them a maternal purification. Insects are equally fond of it, and repeatedly lick one another. By the same means they free their eggs or pupæ from dirt. Every one must also have witnessed, again and again, the scrupulous care with which many animals *wash* themselves. Birds are very fond of this practice, and perform the operation with a skill which evidently manifests that the instinct is heaven-taught. To get a mind-drawn picture of this feat, let the reader think of the maneuvers of a duck at a pond, or the more stately performance of a swan in a stream.

"One of the most curious illustrations our subject admits of was discovered by the talented entomologist before mentioned. It is a special apparatus for cleaning a very peculiar insect. At the bottom of a hole near an old tree Mr. Rennie found a curious grub, which he had never seen before. Taking it home, with a few small snails found in the same place, and watching the creature, he found it employed in a very anomalous manner. Its tail was turned up, and bent over its back, and every now and then removed again. For some time the object of the creature in this occupation was a complete mystery. At length the tail was examined, and the most singular apparatus was there found. In shape it was somewhat like a shaving brush: under the microscope it was found to consist of a double row of white cartilaginous rays which were retractile at the will of the creature, like the horns of a snail. In the interspace was a funnel-shaped pocket, which turned out to be a sort of little dust hole. Now this was its manner of operation; the tail was bent up over the back, and applied to any part of the insect's body; the creature then caused the rays to retract, so as to make the whole act somewhat like a boy's sucker, thus drawing off every particle of dust and dirt from its glossy skin. This done, they were stored up in the little pocket until it was quite full, and then the insect, by a vermicular motion of the same instrument, caused the collected matters to be expelled in the form of a little pellet, which it was careful to deposit out of the way,

"Not only are animals commanded by the author of their being to pay this regard to their personal cleanliness, but the homes of many among them are patterns of neatness and order. How often may we be amused at the diligence of the spider in keeping her net clear of the smallest particle of dirt! What lines will she not cut away and lay down again to secure this end! What a miracle of skill and neatness is a bird's nest, and how assiduously the parent birds remove every impurity from it! Even the proverbial filth-lovers, swine are uncommonly particular in their homes; for it is well known that no creature is so anxious to have a clean and comfortable bed. And very probably the dirt-encasing gambols of these animals are to be excused on the score of an irritating cutaneous affliction, or are intended to resist the stings of insects. Let us hope as we close this short article, that the lessons it is calculated to convey will not be forgotten. Let our poorer classes take just shame to themselves to be alone in their filth. While every domestic animal teaches wisdom, and while all creation exhibits the same pervading principle, will they be content to run the risk of opposing a plain precept of nature? Theirs is not all the blame, when we remember that even statesmen are only just alive to this oldest of all truths, coeval with the very institution of the present scheme. When it has been our lot to visit dirty habitations, and when we remembered the wide-spread lesson taught us in creation, often have Heber's words risen to recollection with a sigh, reminding us

'That only man is vile.' "

We hope that these valuable and interesting truths will not only show the importance of cleanliness, but conduct to the observation of other natural processes scarcely inferior in value and interest. Of all hygienic measures for the prevention of cholera none are as useful and essential as cleanliness, and every possible method that may enforce this on the mind, will do much towards the mitigation of human suffering. If the laws of cleanliness be strictly enforced, the laws of malaria would be of small importance. Cleanliness in the person, in the habitation, and in all contiguous grounds is of the last importance as a preventive measure against cholera, and an extensive array of other diseases. Cleanliness of the house and grounds guards against those impurities that produce the long catalogue of malarious diseases. Cleanliness of the person, under the influence of daily ablutions with clean water does much, not only towards the prevention of many diseases, but for the mitigation of those that are inevitable. Sir Astley Cooper bears impressive testimony to this fact in his lectures. He says, that by daily attention to the spunging of his body with cold water, under all the severe exposures of a student's life in the damp dissecting rooms of London, and those arising from an arduous and extensive practice of his profession, he had passed thirty-years without the sickness of an hour, and his health continued good almost to the termination of his life. Can we then be too strenuous in our exhortations on the importance of this great sanitary measure? Could we well have said less than we have upon a subject that guards our health and protects us at small cost from a series of measures that are troublesome, expensive, complicated and partial in their action? The inspiration of the Bible is most impressive on this subject, and is forcible in its denunciations of uncleanness and pollution. The inspiration of observation showed Mahomet the value of cleanness, and he made it a prominent part of his institutions. Medical observation has recorded the result of his laws. Wherever cholera prevailed in the dominions of the Crescent, it was observed that the Musselmen, who are strict in their observance of the precepts of the Koran in regard to ablutions escaped the disease, while those who were less strict in their reverence for them suffered severely.

If, however, from causes beyond individual control, it is found impossible to create such a state of cleanliness as to give perfect confidence, we must turn our attention to the laws of malaria, and from them establish such sanitary measures as will perfectly arrest the march of the disease. No half-way measures must be thought of. The attempt to guard against malaria must be perfectly thorough, or through some neglected crevice we shall feel the approaches of the unwelcome guest, while we are lulled into a false security.

The various physicians throughout the world, and nearly all Boards of Health, upon the testimony of physicians on the spot, have borne strong testimony to the value of drainage as a means of preventing cholera, and its kindred diseases. If what was intended for a drain becomes a stagnant ditch, that which was made for the promotion of health, becomes a source of pestilence. The sluggish stream that moves its lazy waters along sedgy banks is a source of evil, while the bubbling brook that runs merrily over its pebbly channel as it moves its limpid waters towards the ocean, is a purifier, and thus becomes a source of health. No stagnant pools of water should be permitted to exist in the neighborhood of human habitations. If their exhalations do not rise at all times into that malignancy that cuts the cord of life in a few brief hours, they do not the less certainly undermine the foundations of vitality, and slowly and surely they "break the golden bowl, and the pitcher is broken at the fountain." Yet how often do we see the pool at the door, permitted year after year to sow its seeds of pestilence in the family, giving rise to a long train of evils that is the result of a violation of the plainest dictates of common sense? The philosophic and honest physician will always feel himself bound by his duty to that profession, whose first great object is to do good, to urge the prompt removal of all sources of malaria. Thorough drainage removes one of the most essential elements of that agent of disease and death, and becomes thereby a most important sanitary measure. We cannot control solar temperature, nor the decomposition of vegetable matter, but we can deprive the latter of that amount of moisture that makes its decomposition pernicious. There are times when the conjunction of solar heat and moisture for action upon masses of filth is so apparent that if attention were directed to the approaching danger, all the evils might easily be diverted. Thus, for instance, if, in 1833, the people of Lexington, Ky., when a high temperature with an unusual amount of rain showed itself, had improved the drainage so as to carry off rapidly the water that formed in pools in the flat part of the town, or had covered with lime the masses of filth that had been accumulating for years in that portion of the city, nearly all the disasters of cholera might have been averted. The same facts were developed in New Orleans in December, January, and March and April of the present season. These are instructive lessons that should not be forgotten for the future. Defective drainage in a town is an evil that cannot be too speedily removed. The pernicious effects of malaria are not confined to sweeping and fearful visitations of cholera;—the latter are fortunately but occasional exhibitions of the evils of that morbific agent; but wherever malaria is permitted to form it is a continual source of disaster. The liver and spleen become effected under its mildest production, and life is made an insupportable burden. In healthy countries the average of human life is fifty years, but in malarious regions this average dwindles down to twenty-five years, in Holland, to twenty years, in portions of France, Spain and Italy, and to ten years in parts of Africa, and in the East and West Indies. The ravages of cholera over the world have caused a great deal of investigation into this subject and lasting good has been done in the improvements that have taken place in many regions that have been scourged with the pestilence. But it should never be forgotten that the presence of filth and moisture may be productive not only of cholera, but of various other forms of disease that are scarcely less destructive to life, but which are slower in their ravages. These facts should impress the mind with the importance of cleanliness and drainage as sanitary agents.

In order to understand thoroughly a proper system of sanitary measures for protection against visitations of cholera it is essential to have an intimate acquaintance with the laws of malaria, and to them we shall now address our remarks.

Let us not be misunderstood so far as to have it supposed that we think rains essential to the production of the requisite moisture for malaria. We wish to present the subject in all its possible forms, and as rain is the most general furnisher of the necessary moisture we have presented it prominently. But rain may be a protective agent sometimes against the effects of malaria. The pernicious effects of malaria are felt in the Campagna di Roma during excessively dry weather, and heavy rains would prevent them, by washing out the pestilent ditches and drains that are so injurious to life, or by covering up the decaying vegetable matter with water, thereby protecting it from solar heat. Thorough dryness prevents the decomposition of all kinds of matter, and this important truth should not be neglected in the construction of a system of sanitary measures. But there are cases where it would be equally important to remember that excessive moisture also prevents putrefaction. This latter fact explains the reason why malarious disease sometimes affects the inhabitants on the crown of a hill, while those residing in the flat below are exempt. The water rolls down the sides of the hill and accumulates below in sufficient quantities to prevent the decomposition of vegetable matter, while enough remains on the crown of the hill to produce the pernicious effect.

The topography of a place has much to do with its relation to malaria. In a very wet summer extensive districts become unhealthy that are uniformly healthy in dry summers, except in the vicinity of swamps, ponds or water courses, and these unhealthy localities of a dry summer become healthy points in a wet one, when the districts that are healthy in a dry summer become sickly if that season is a wet one. These facts were established on an extensive scale, in this country, in 1822 and 1823. Dr. Cooke says that in the excessively dry summer of 1822 the valley of the Shenandoah in Virginia was generally healthy. The few places that suffered were near marshy grounds, or rivers, which in that arid season were almost dried up, leaving extensive flats covered with an abundance of the vegetables which grow in water, exposed to the action of the sun. The highlands about the Blue Ridge were healthy, and the parts about the marshy borders of rivers alone suffered. In Pennsylvania, about Harrisburg, July was wet and sickly, but the weather becoming hot and dry removed the moisture and stopped the sickness. The summer was so dry that the Ohio river became lower than it was ever known before. The water was nearly stagnant, "resembling a big lake more than a river, and covered with a mucous scum or froth. Disease was confined to the marshy districts." Louisville, Ky., was then a town of swamps, and was the scene of a desolating sickness. These are mere glimpses of the state of things that generally prevailed in the dry summer of 1822, but the summer of 1823 being a wet one, made a great change in the operations of Malaria. "The rains were excessive in Pennsylvania, Maryland, Virginia, Ohio, Mississippi and Alabama, and the autumnal epidemic was very severe in all these states. At Natchez the year 1816

was very dry and healthy, 1817 wet and sickly, 1818 dry and healthy, and 1819 wetter and more sickly than in 1817."

These facts are sufficient to show the varying phases of heat and moisture that must be taken into consideration in the successful execution of sanitary measures.

Masses of vegetable matter should never be permitted to decompose in the neighborhood of habitations. They may decompose in hot, dry weather without any evil effect, but if even small quantities of rain fall in this state of things a sudden and alarming pestilence may spring up. This was the case in New Orleans in December 1848 and January of the present year. There were great accumulations of vegetable filth, the thermometer ranged at 80° and 85°, and when the requisite moisture was supplied an alarming pestilence of cholera showed itself. This continued until cold weather checked it, and now, since the weather has become warm again, the cholera has resumed its ravages. In 1832, in the early part of the fall we had no rain in Louisville, and September was cool, but the first four days of October were very hot, attended with rain, and on the 4th day of that month the cholera commenced its ravages in this city. There had been sporadic cases in July and August, but in October the only faint resemblance we have had to epidemic cholera in Louisville showed itself.

Lancisi noticed the evil effects of clearing timbered land in his day, and numerous facts since that time have corroborated his observation. The city of Philadelphia was once protected from the exhalations of some neighboring marshes, by a forest growth between the city and the marshes; when this timber was removed, the city was afflicted with pestilences of different grades, and yellow fever among them, until the marshes were leased out, drained, and filled for the purpose of converting them into an immense vegetable garden. This changed the malarious topography, and Philadelphia has never been seriously desolated since with yellow fever. The Cholera in Philadelphia, in 1832 was mainly confined to the malarious district in the neighborhood of the Schuylkill. The season was then, as in Lexington, Ky., the year after, propitious for the production of malaria. Sydenham noticed in his day, when he was altogether ignorant of the cause of cholera morbus, that it came in the summer as certainly as swallows did. In other words it made its appearance at that season of the year when malaria is most certainly produced. The meteorological tables kept by Cleghorn, in the island of Minorca, from 1744 to 1749 very conclusively show that cholera morbus is an effect of the cause that produces remittent, intermittent and continued fever, dysentery, &c. Dr. Jackson, quoted, by Dr. Cooke, in allusion to an epidemic yellow fever which prevailed in Philadelphia in 1820, says: "in the past summer and autumn diseases assumed the general symptoms which they possessed in the former epidemic of 1793, 1797, and 1798. *Cholera morbus and infantum were very prevalent;* bilious and remittent fevers from which our city had been for several years nearly exempted, were common diseases; and dysentery, which had become a rare disease in Philadelphia, was of frequent occurrence, and very difficult to manage." The occurrence of dysentery, intermittent fever and other forms of malarious disease in connection with cholera is almost universal, but is often unrecorded because of the greater importance attached to cholera. The coincidence of these forms of malarious disease, during the prevalence of cholera, was noticed in Louisville in 1832, and in 1833, in Lexington, Simpsonville, Ky., in Palmyra, Mo., and Dr. Halphen, a thorough contagionist, incidentally mentions the fact, that during the prevalence of cholera in New Orleans, even the cases of fever and ague, had cholera symptoms. The reader of our former sketch will remember the statements we collected from many sources, giving descriptions of the localities of cholera. The following statement of Dr. Cooke, when speaking of cholera infantum, shows how perfect the identity is in the localities of what is called Asiatic cholera and cholera morbus and infantum. Dr. Cooke says: "In cities, those parts most favorable to the production of fevers, such as narrow dirty alleys, and filthy suburbs in the neighborhood of marshy inlets, brick-yards, standing water produced by regulating streets, &c., are also most productive of cholera infantum." If he had been giving the topography of cholera itself, he could not have given a more accurate description. No matter what the situation may be, whether high or low, wherever water may stand, malaria may be produced, and wherever that is the case cholera may show itself. We are not yet well enough acquainted with all the minute matters that are concerned in the production of malaria, to say with certainty what particular form of disease its presence may produce; attempts have been made to give specific names to the different kind of malaria that produce the infinite variety of diseases ascribed to it, but such knowledge is quite useless until we know the nature of the changes. We can tell with certainty that malarious disease is about to commence its operations, but we do not know what form it may assume until the disease developes itself. When we know what the circumstances are that are essential to the formation of malaria, we know enough to prevent its operations, and to this important point the philosophic physician should address himself.

But a residence immediately contiguous to a source of malaria is not by any means essential to its action. Persons at considerable distances from the origin of malaria may be severely affected by it. It is carried by wind and fogs to considerable distances. The latter medium o transportation has carried malaria so frequently, that at one time fogs were believed to be the cause of remittent and intermittent fevers, until it was noticed that the dense and long continued fogs of Newfoundland did not give rise to any diseases of this description. The fishermen live for months in the fogs formed by the condensation of the vapors arising from the gulf stream in the neighborhood of Newfoundland, and they are not affected with agues, autumnal fever, nor any of the forms of malarious disease.

It has not been accurately determined how far malaria, sufficiently strong to produce disease, may be carried by the wind. Malaria cannot rise perpendicularly more than forty feet, but it may be carried some distance up the surface of a hill. The convent of Camaldoli is often referred to as an instance in point. That convent is about three miles from the malarial lake Agnano; it has no local source of malaria, yet malarious disease occasionally shows itself among the inmates, and the malaria is properly referred to the lake Agnano. While the English troops were suffering on Walcheren island, persons on board of the ships lying between two and three thousand feet from the shore escaped. A similar result has often been noticed in various parts of the world. This fact shows that malaria cannot pass any great distance over water, and in favorable positions may prove useful as a sanitary measure.

The tenacity with which cholera clings to damp situations, and the fondness of malaria for such places, if the expression is allowable, is conclusive proof of the malarious origin of cholera. A moist air will convey malaria a greater distance than dry air will; and this fact is offered as an explanation of certain phenomena in malarious diseases. It has been noticed sometimes that malaria attacks a hilly situation, while the plain is exempted, and this has been observed in the history of cholera. The explanation is, that a hill attracts moisture from the clouds, which affords a pabulum for the malaria, while the dry air of the plain destroys it. The application of this fact to some of the features of cholera throws light upon a seeming paradox.

The conveyance of malaria by the winds is an old and well established fact. It first roused the attention of Lancisi to the subject, and since his day the fact has been abundantly corroborated. Residents on the leeward side of a marsh are more likely to suffer from malaria than those on the windward side. And the character of the wind too has something to do with the evil. A south wind, as a general rule, is a better conductor of malaria than a north wind is. There is no doubt of the fact, that the south wind conveys it in an active condition to greater heights than the north wind does. Captain Smith, quoted by Dr. Elliotson, in his "Statistical table of Sicily," mentions seventy-six unhealthy towns and villages, and thirty-five of them are situated on hills or declivities, and at a great distance from the marsh. Dr. Elliotson adds: "When the wind blows from the south, being a warm wind, it has a tendency to ascend; and it is supposed that the southern wind—blowing over a marsh, and tending upwards by its temperature—affects high towns, while the colder northern wind does not affect those houses situated on the other side of the swamps, though placed equally high."

Another singular fact noticed in cholera, and which has surprised many, is, that in many instances one side of a street is assailed by the disease, while the other side is free from it. This has been noticed repeatedly in cholera, and is forcibly stated by Dr. John Bell, in his remarks on epidemic cholera. The fact has been noticed all over the world, and was strikingly exhibited in Augusta, Ga., in 1839. In that city, one side of a street was severely ravaged, while the other side was entirely exempted from the disease. Even in camps this singularity has been noticed. In this feature of cholera, we have another strong corroborative proof of the malarious origin of cholera. This singularity has been often noticed in various forms of malarious disease, and is often exemplified in agueish attacks. Observers of natural phenomena have noticed, says Dr. Elliotson, that dew and frost show the same singularity. They will spread to a particular spot or bush, and none will be found beyond them. This, too, must be the case with malaria—it is conveyed to a certain spot, exerts its influence there, and is restricted there. The minute and extensive observations that are in the course of accumulation on the subject of meteorology will yet clear up all these difficulties, and we must hold fast to the facts we have, in order to enjoy the advantage of those that may be gathered hereafter.

Having thus explained the sources of malaria, a knowledge of which may lead easily to methods for avoiding its disastrous effects, we shall now turn our attention to other features of the subject, which are scarcely less important than a knowledge of the sources of this agent of danger. Position has much to do with the dangers of malaria. It attacks with more violence those who sleep near the ground, than it does those who lodge in upper stories. It is highly probable that malaria is much heavier than atmospheric air; hence a reason for its action upon lodgers in lower rooms. We have often, in Louisville and in Jefferson county, seen striking illustrations of the law; the most violent forms of fever are found in the lower apartments, milder forms in the second story, and no trace of malarious disease in the third. In 1832 and in 1833, when the cholera was in Louisville, we took advantage of this fact with a number of families living in houses with basement stories. We induced them to remove their negroes to the attics of their dwellings, and not an attack occurred among the negroes thus protected, while the servants living in adjoining houses, and who slept in the basement rooms, died with the disease. This is a valuable truth that should be remembered in regard to all malarious disease. Whether malaria clings to the earth because of its weight, or from some peculiar attraction by the earth's surface, or because the lower strata of air are more moist than the upper ones; the fact that its noxious influence does not rise to any great perpendicular height, is one that is well established, and should be acted upon during a malarious season. Another important law is, that malaria attacks with greatest potency at night. Hecker bears testimony to the fact that it was noticed in the epidemics of the middle ages, that the cause of those dreadful scourges acted with most power in the night. The physicians of that day, though utterly ignorant of the nature of the cause of those epidemics, noticed the fact, we are upon, so well that they deduced a prophylactic measure from it, and they warned the people against the noxious qualities of night air at particular hours. The fact thus noticed has been abundantly proven since that time. Dr. Elliotson quotes from "Lind on Hot Climates," the following facts: "When the ship Phœnix lay at anchor at St. Thomas's, two hundred and eighty men, constituting nearly all the ship's company, went on shore in parties of twenty or thirty, and rambled about the island, but none of those who did not remain on shore at night suffered; while none of those who slept on shore escaped sickness, and only three survived." The same author says: "At Batavia, a boat belonging to the 'Medway' was actually manned three times—every one having perished; and simply from the men having to attend duty on shore every night." The next year similar events happened to the same ship, at the same place, where "she lost eight men out of ten, who had imprudently remained *all night on shore*, while the rest of the ship's company, who, after spending the greatest part of the day on shore, always returned to their vessel before night, continued in perfect health." The force of the law we are illustrating is seen in the fact that all forms of violent malarious disease are most apt to attack at night. A large majority of cholera cases commence in the night. The greater potency of malaria at night does not probably arise from any inherent quality in it, but is rather owing to the fact that all the sentinels of vitality are then asleep at their posts, and the powers of life are then less guarded than during our hours of active life. It is questionable whether malaria acts with any more power at night, on a person who keeps in motion, than it does during the day. Hence the reason why physicians are more exempt from malarious disease than any other class who have the same amount of exposure. The duties of their profession carry them into the focus of danger, but the active life they are compelled to lead guards them against the morbific effects of malaria. Even in the pestiferous Pontine marshes, where no stranger can escape if he lies at rest at night, active exercise is found to be of great service. Dr. Watson says: "The reapers in the 'Campo Morto,' a well known part of the Maremma, are permitted to sleep for two hours at noon. They do so at that time without danger; but when the dews of evening have fallen down upon the earth, which serves them for their bed, it is then that the poison puts forth its most deadly power." From all the facts we have found, this seems to be the law:—malaria has perhaps most power at night, and in a state of complete rest the body is more susceptible to the influence of the poison than when in active motion.

But motion in the open air of a malarious district should be avoided *early in the morning*. Abundant experience in this country has established this as an important measure of security, and it is recommended by experience that those persons whose avocations carry them out early in the day, should invigorate the system by some nourishing food, and, as a general rule, coffee and bread will be found to answer the purpose. We have seen this principle very usefully employed in cholera districts.

It follows, as a matter of course, that if malaria is mixed with the atmosphere, it will travel with it, and the movement of malaria, by currents of air, from its source to a distant point, has been frequently noticed. An immense volume of facts might be written in proof of this. The instance of the convent of Camaldoli has been already referred to, and Dr. Ferguson has given some striking examples in cases that occurred at the island of Dominica. His facts are interesting, but his inferences are very wild. Dr. Ferguson says, that there are two hills at the port, Prince Rupert, which are joined to the main land by a flat and very marshy square isthmus to *windward*, of about three quarters of a mile in extent. The inner hill is of a slender pyramidal form, and rises from a narrow base nearly perpendicular, above and across the marsh from sea to sea. The outer hill is a round-backed bluff promontory, which breaks off abruptly in the manner of a precipice above the sea. Between the hills runs a very narrow clean valley, where all the establishments of the garrison were originally placed," (and this did not much improve the cleanliness of the valley); "the whole space within the peninsula being the driest, the cleanest, and healthiest surface conceivable. It was speedily found that the barracks in the valley were very unhealthy; and, to remedy this fault, advantage was taken of a recess or platform near the top of the inner hill, to construct a barrack which was completely concealed by the crest of the hill from the view of the marsh on the outside, and at least three hundred feet above it; but it proved to be pestiferous beyond belief. No white man could live there, and it had to be abandoned. At the time this was going on, it was discovered that a quarter which had been built on the outer hill, on nearly the same line of elevation, and exactly five hundred yards farther removed from the swamp, was perfectly healthy; not a single case of fever having occurred in it from the time it was built." In the case of the unhealthy hill, the malaria was carried up the face of the hill, and rolled in a torrent down upon the garrison, hence the fatality. These facts show the importance of ascertaining the direction of the prevailing winds during the malarious season of the year, in determining the location of a dwelling. But if this is already fixed, a protective measure may be found in the interposition of a screen of living vegetation between the marsh and the dwelling, and while trees are growing, resort may be made to the planting of annuals that grow luxuriantly. A screen of vegetation is a potent agent for arresting malaria.

In numerous histories of cholera we have seen the statement made, that the disease commenced its ravages immediately after a fall of rain. In some instances the disease appeared in a few minutes after the rain. Dr. Elliotson quotes Lind and Mungo Park in proof of the fact that "malaria is precipitated by rain. Park says that, on one occasion the rain had not begun more than three minutes before many soldiers seemed drunk, and fell asleep; while others vomited; and he mentions that, in a storm, he himself felt disposed to sleep, and could not help it, although he was on damp and, therefore, dangerous ground."

The use of lime about dwellings is another excellent mode of arresting the effects of malaria. It should be used with much profusion.

We have thus carefully traced the cause of cholera, and from a most minute and careful examination of the history of the disease in every part of the world, we are as certain of its malarious origin as we are that intermittent fever is the result of malaria. We speak from personal knowledge, when we say that we have seen cholera faithfully obey every law of malaria; and we have never known it to disregard any one of those laws. We have seen the disease controlled in its march by those means that disarm malaria of its power; and we have never seen this done by any other method. Hence the value of the minute acquaintance with the whole subject of malaria, which we have endeavored to inculcate in these papers. In portraying the subject, we have generally followed the paths of Drs. Elliotson and Watson, but we have carefully abstained from all speculations, and have endeavored to confine our remarks exclusively to useful matter. This has been a labor of no ordinary character, for, in order to speak the truth respecting the cause of cholera, we have not only faithfully read every history within our reach of the disease in every part of the world, but we have studied the topography of every place where it has appeared, and have gathered all the meteorological information it was possible for us to command. Why we have thus labored on this subject is well expressed in the following remarks in the July number of "The British and Foreign Medico-Chirurgical Review." The reviewer thus eloquently and impressively addresses himself to medical men:

"The true philosophy of the science of medicine is the knowledge of the causes of disease. Or, if these causes be too subtile and refined for our gross senses, it is the knowledge of the several conditions, external or internal to the body, which give those causes power. In the future history of medicine, we shall see men returning to the principles promulgated by its earliest founders. They will perceive that the treatment of the fully formed disease is at the same time the most difficult, and the least useful part of their noble profession. They will learn to arrest the evil at the fountain-head, and not to dam the current swollen by a thousand tributaries. And if the principles which we have analyzed in this article be correct, it will not be the least triumph of this philosophy, that it has indicated the true mode in which the great epidemic of our time can be most easily and most effectually controlled. It bars out the disease, not with quarantines and cordons sanitaires, but with a cleanly people and uncontaminated air. The evil which springs from the bosom of nature only needs for its removal an observance of the rules which nature herself reveals."

In the article to which these admirable remarks are a peroration, there is no attempt to trace the malarious origin of cholera, yet the writer conclusively establishes the fact, without intending to do so, that malaria is the cause of cholera. We have abundantly proven this point, and we repeat, that in the whole history of the disease, there is not a fact that mars the proof.

We shall close this department of our subject by calling attention to the suggestions made by the able body of gentlemen who were appointed by the poor law commissioners of London, to investigate the condition of the metropolitan poor houses with reference to cholera. We feel pleased with the fact that the gentlemen thus appointed had no idea of malaria in connection with cholera, and this gives their suggestions peculiar force. If they had had the malarious theory, their sanitary plan would have been open to the objection, that it was made to conform to their theory. As it is, the recommendations are the result of an immense deal of observation, experience, and testimony from many quarters, portions of which we quoted in our April number. The suggestions to which we allude have been adopted in London, and have been found remarkably successful and accurate. The editor of the London Lancet says: "these simple measures are worth all the nostrums or specifics which have ever been vaunted for the cure of Asiatic cholera." The quotations we make are exactly conformable to the laws of malaria, and show a most triumphant proof of the accuracy of the doctrine of the malarious origin of cholera. Here are the sanitary regulations of London, based upon one of the most minute investigations that ever was made into the circumstances attendant on an epidemic disease:

"Let every impurity, animal and vegetable, be quickly removed to a distance from the habitations; such as slaughter houses, pig sties, cesspools, necessaries, and all other domestic nuisances."

We do not believe that animal putrefactions are ever connected with epidemic diseases, but there can be no objection to their removal from habitations.

"Let all uncovered drains be carefully and frequently cleansed.

"Let the grounds in and around the habitations be drained, so as effectually to carry off moisture of every kind.

"Let all partitions be removed from within and without habitations which unnecessarily impede ventilation.

"Let every room be thrown open for the admission of fresh air; and this should be done about noon, when the atmosphere is most likely to be dry.

"Let dry scrubbing be used in domestic cleansing, in place of water cleansing.

"Let excessive fatigue and exposure to damp and cold, especially during the night, be avoided.

"Let the use of cold drinks and acid liquors, especially under fatigue, be avoided; or when the body is heated.

"Let a poor diet, and the use of impure water in cooking or for drink, be avoided.

"Let the wearing of wet and insufficient clothing be avoided.

"Let a flannel or woolen belt be worn around the belly.

"N. B. This has been found serviceable in checking the tendency to bowel complaint, so common during the prevalence of cholera. The disease has, in this country, been always found to commence with a looseness in the bowels, and in this stage is very tractable; it should, however, be noticed, that the looseness is frequently attended by pain or uneasiness: and fatal delay has often occurred from the notion that cholera must be attended with cramps. In the earlier stage here referred to, there is often no griping or cramp, and it is at this period that the disease can be most easily arrested.

"Let personal cleanliness be carefully observed.

"Let every cause tending to depress the moral and physical energies be carefully avoided; let exposure to extremes of heat and cold be avoided.

"Let crowding of persons within houses and apartments be avoided.

"Let sleeping in low or damp rooms be avoided.

"Let fires be kept up during the night in sleeping or adjoining apartments, the night being the period of most danger from attack, especially under exposure to cold or damp.

"Let all bedding and clothing be daily exposed during winter and spring to the fire, and in summer to the heat of the sun."

But our diminishing space admonishes us to arrest any further investigation at this time. We had hoped to be able to show how conclusively the proof establishes the fact that what is called Asiatic cholera is but a grade of cholera morbus, and, that they are not any more distinct diseases, than confluent and distinct small pox are. We hoped also to enter upon an investigation of the proper principles of treatment for cholera, and to make an examination of some of the prominent methods in vogue. We have room only, however, to urge upon our friends to be guarded against the extraordinary opiate treatment urged by Dr. Hawthorne. That gentleman's pamphlet has been republished and extensively distributed in the west and south, and must exercise a disastrous influence wherever its principles are followed. It is utterly impossible that such doses of opium, as are recommended in that treatise, can be given without inflicting serious injury. T. S. B.

